# Analysis of biochemical parameters and tumor markers in Cushing’s disease and ectopic adrenocorticotropic hormone syndrome

**DOI:** 10.7717/peerj.21677

**Published:** 2026-10-02

**Authors:** Linjia Guo, Yingqi Liu, Saijia Li, Xinyue Yu, Kewei Wang, Fei Yan

**Affiliations:** Department of Endocrinology and Metabolism, Qilu Hospital of Shandong University, Jinan City, Shandong Province, China

**Keywords:** Cushing’s disease, Ectopic ACTH syndrome, Diagnosis, Tumor markers, ROC curve, Midnight cortisol, ACTH

## Abstract

**Purpose:**

To compare clinical characteristics, biochemical parameters, and tumor markers between patients with Cushing disease (CD) and ectopic adrenocorticotropic hormone (ACTH) syndrome (EAS), identify potential biomarkers, and optimize their diagnostic differentiation.

**Methods:**

A single-center retrospective study was conducted in 75 patients with ACTH-dependent Cushing syndrome (51 CD, 24 EAS) admitted between January 2006 and April 2025. Differences in biochemical indices, tumor markers, and hormone levels were analyzed. Receiver operating characteristic (ROC) analysis was used to evaluate diagnostic performance and determine optimal cut-off values.

**Results:**

EAS patients were significantly older at diagnosis than CD patients. Fasting glucose was higher, while serum potassium and albumin were lower in the EAS group. Non–small cell lung cancer-associated antigen, carcinoembryonic antigen (CEA), cancer antigen 15-3 (CA15-3), and cancer antigen 19-9 were markedly elevated in EAS, whereas pro-gastrin-releasing peptide (ProGRP) and neuron-specific enolase (NSE) showed no significant differences. Serum cortisol and plasma ACTH levels at all time points and after dexamethasone suppression were significantly higher in EAS. ROC analysis of the product of midnight serum cortisol (μg/dL) and plasma ACTH (pg/mL) divided by 1000 (“Midnight Cort × ACTH/1000”) showed an area under the curve (AUC) of 0.902 for identifying EAS, with a cut-off value >2 (sensitivity 83.3%, specificity 84.3%). Its diagnostic accuracy was comparable to that of the high-dose dexamethasone suppression test (HDDST).

**Conclusion:**

EAS patients were older and exhibited more severe metabolic abnormalities than those with CD. Elevated tumor markers may suggest ectopic ACTH secretion. The “Midnight Cort × ACTH/1000” index provides high diagnostic value and a simple, convenient method for differentiating EAS from CD.

## Introduction

Cushing syndrome (CS), or hypercortisolism, results from chronically elevated cortisol levels caused by various etiologies. The global incidence is estimated at 1.8–4.5 cases per million people annually, with a prevalence of 57–79 cases per million (Giuffrida et al., 2022; Wengander et al., 2019; Hakami, Ahmed & Karavitaki, 2021; Gadelha et al., 2023). Adrenocorticotropic hormone (ACTH)-dependent CS accounts for more than 80% of all cases (Hakami, Ahmed & Karavitaki, 2021; Lacroix et al., 2015; Valassi, 2022), and Cushing disease (CD) represents 80–90% of ACTH-dependent forms (Giuffrida et al., 2022; Fleseriu et al., 2021). Ectopic ACTH syndrome (EAS) constitutes 10–20% of ACTH-dependent cases (Valassi, 2022; Hayes & Grossman, 2022; Young et al., 2020). The lung is the most frequent tumor site, where pulmonary neuroendocrine tumors represent approximately 25% of cases, followed by small cell lung cancer, which accounts for about 20%. Other causes include thymic and pancreatic neuroendocrine tumors, medullary thyroid carcinoma, and pheochromocytoma or paraganglioma (Hayes & Grossman, 2022; Young et al., 2020; Isidori & Lenzi, 2007). CD and EAS exhibit overlapping clinical features, including central obesity, hypertension, impaired glucose metabolism, dyslipidemia, osteoporosis, and electrolyte disturbances (Valassi, 2022). The primary treatment for CD is transsphenoidal surgery (Hakami, Ahmed & Karavitaki, 2021), whereas management of EAS involves localization and resection of the ectopic tumor, sometimes combined with chemotherapy or targeted therapy (Young et al., 2020; Nieman et al., 2015; Seastedt et al., 2021). Diagnostic errors or uncertainty may delay appropriate treatment or result in unnecessary surgical intervention.

According to the 2025 Chinese Expert Consensus on the Diagnosis and Treatment of Cushing’s Disease, the high-dose dexamethasone-suppression test (HDDST) is recommended as a first-line routine assay for CD-EAS differentiation. The standard protocol involves oral administration of 8 mg dexamethasone daily for two consecutive days. Suppression of 24-h urinary free cortisol (24 h UFC), 24-h urinary 17-hydroxycorticosteroids (24 h 17-OHCS), or serum cortisol by more than 50% compared with baseline indicates CD; otherwise, EAS is suggested. However, HDDST has marked constraints: its sensitivity for CD is only 60–80% (Elenius, McGlotten & Nieman, 2023; Ceccato et al., 2023), with 20% of CD patients showing no suppression response and false suppression occurring in well-differentiated EAS such as bronchial carcinoid (Tsagarakis et al., 2002). Its cumbersome protocol, high requirement for patient adherence, and risks of exacerbating metabolic disorders and infection further limit its application, and major international guidelines no longer recommend HDDST as a routine differential tool (Fleseriu et al., 2021). The corticotropin-releasing hormone (CRH) stimulation test, recommended by international guidelines, has a sensitivity and specificity over 90%, outperforming HDDST (Elenius, McGlotten & Nieman, 2023; Ceccato et al., 2023). However, no CRH diagnostic reagent has been approved in China, with limited access and high costs precluding its routine clinical use. Bilateral inferior petrosal sinus sampling (BIPSS), the recognized diagnostic gold standard with nearly 100% accuracy for CD (Wang et al., 2020), is restricted by its invasive nature, high technical requirements, and poor accessibility. Pituitary MRI yields ~40% false negatives for microadenomas (Adam et al., 2017), and 10–20% of EAS tumors are occult on conventional imaging. Therefore, the development of a simpler and safer alternative diagnostic approach holds significant clinical importance.

Few studies have examined differences in biochemical indicators and tumor markers between CD and EAS. Limited evidence suggests that certain neuroendocrine tumor markers, such as ProGRP, may be elevated in some patients with EAS (Valassi, 2022). The diagnostic value of tumor markers commonly used in lung and gastrointestinal cancer screening, including CA15-3 and CA19-9, has been rarely evaluated in the context of EAS differentiation. The present study aims to compare general clinical characteristics, biochemical parameters, and tumor markers between patients with CD and EAS, identify potential biomarkers, and improve the diagnostic workflow.

## Materials and Methods

### Study subjects

A single-center retrospective study was conducted involving 75 patients with ACTH-dependent CS who were hospitalized in the Department of Endocrinology and Metabolism, Qilu Hospital, Shandong University, between January 2006 and April 2025. This study was performed in line with the principles of the Declaration of Helsinki. Ethical approval was granted by the Research Ethics Committee of Qilu Hospital, Shandong University on 26 January, 2022 (Ref: KYLL-202111-042). The requirement for informed consent was waived by the same committee due to the retrospective nature of the study and the use of anonymized data. Patients were classified into two groups according to etiology: a CD group (*n* = 51) and an EAS group (*n* = 24). In addition, 17 cases met the diagnostic criteria for occult ACTH syndrome, as no identifiable tumor lesion was detected *via* comprehensive biochemical and imaging workup. Given that the final etiology remained unconfirmed and the patients did not meet the CD/EAS grouping criteria for this study, they were excluded from the final analysis. Inclusion criteria: (1) Diagnosis of ACTH-dependent CS based on the 2025 Expert Consensus on Cushing Disease. Standard differential diagnostic workflow for CD and EAS in our center: After biochemical confirmation of ACTH-dependent CS, all patients underwent initial 3.0T dynamic contrast-enhanced pituitary MRI. CD was diagnosed in those with typical PitNET ≥6 mm, combined with HDDST and biochemical features. Bilateral inferior petrosal sinus sampling (BIPSS) was performed for definitive diagnosis in patients with negative MRI, lesions <6 mm, or discordant imaging-biochemical results. All patients received enhanced chest-abdominal CT and somatostatin receptor scintigraphy for ectopic tumor screening; (2) Grouping criteria: CD group: Patients who underwent transsphenoidal surgery with postoperative immunohistochemical staining positive for ACTH; EAS group: Patients with ectopic ACTH-secreting tumors confirmed by ACTH-positive immunohistochemical staining of biopsy pathology.

### Data collection

Detailed patient information was extracted from the electronic medical record system. Demographic data included age, sex, height, weight, body mass index (BMI), systolic blood pressure, and diastolic blood pressure. Biochemical indicators included aspartate aminotransferase (AST), alanine aminotransferase (ALT), γ-glutamyltransferase (GGT), creatinine, blood urea nitrogen, total cholesterol, triglycerides, low-density lipoprotein cholesterol (LDL-C), high-density lipoprotein cholesterol (HDL-C), albumin, serum electrolytes (potassium, sodium, calcium, phosphorus, and magnesium), and 24-h urinary electrolytes (potassium, sodium, calcium, phosphorus, and magnesium). Tumor markers included neuron-specific enolase (NSE), pro-gastrin-releasing peptide (ProGRP), carcinoembryonic antigen (CEA), carbohydrate antigen 15-3 (CA15-3), carbohydrate antigen 19-9 (CA19-9), and non–small cell lung cancer-associated antigen. Hormone levels included plasma cortisol and ACTH concentrations measured at 08:00, 16:00, and 24:00. Functional tests included results of the LDDST and the HDDST.

### Laboratory testing methods

All blood samples were obtained under standardized conditions. Cortisol and ACTH levels were measured using electrochemiluminescence on a Roche Cobas e601 analyzer. Tumor markers were determined by chemiluminescent immunoassay on an Abbott Architect i2000SR analyzer. Biochemical indicators were assessed by enzymatic methods using a Hitachi 7600 automatic biochemical analyzer. All tests were conducted in strict accordance with standard operating procedures and quality control requirements.

In our study, all ectopic ACTH-secreting tumors in the EAS group were confirmed by ACTH-positive immunohistochemical staining on biopsy or surgical specimens. The primary antibody used was the ACTH antibody reagent (immunohistochemistry) manufactured by OriGene Technologies (Beijing, China) under the brand ZSGB-BIO, with product code ZM-0004. This antibody is a mouse monoclonal antibody (clone 2F6, IgG1 subtype) specifically raised against human ACTH, as clearly stated in the product manual. The clone 2F6 antibody recognizes the mature ACTH molecule and has been validated for use on formalin-fixed, paraffin-embedded human tissue sections. Immunohistochemical staining was performed according to the manufacturer’s protocol using a polymer-based detection system (DAB Chromogen Kit) with appropriate positive and negative controls.

### Statistical analysis

Statistical analyses were performed using SPSS version 30.0 (IBM Corp., Armonk, NY, USA) while Bootstrap sampling analysis and plotting were completed with R language. All measurement data were subjected to the Shapiro-Wilk normality test. Non-normally distributed variables were expressed as medians with interquartile ranges, and normally distributed variables were expressed as means with standard deviations. Differences between groups were analyzed using the independent samples t-test for normally distributed variables and the Mann–Whitney U test for non-normally distributed variables. Categorical data were described as number of cases. The Chi-square test was used to compare categorical variables The skewed distributed variable of Midnight Cort × ACTH/1000 was processed with log10 transformation, after which a multiple linear regression model was constructed by the stepwise method. Receiver operating characteristic (ROC) curve analysis was performed to assess the diagnostic performance of each parameter, calculating the area under the curve (AUC), optimal cut-off value, sensitivity, specificity, and Youden index. Reverse transformation was performed on the HDDST inhibition rate to unify the effect direction. The DeLong test was applied to compare AUCs between indicators, and the Bootstrap method was used for result correction and robustness evaluation. A two-tailed *P* value less than 0.05 was considered statistically significant.

## Results

### General data and biochemical indicators

Among the 24 EAS patients, the primary tumor sites and grades (for NETs) were as follows: bronchial carcinoid (*n* = 10, including NET G1 in six cases and NET G2 in four cases), small cell lung cancer (*n* = 7, classified as NEC), thymic carcinoid (*n* = 1, NET G2), pancreatic neuroendocrine tumor (*n* = 3, NET G2), medullary thyroid carcinoma (*n* = 1), and unknown primary with biopsy-confirmed ectopic ACTH secretion (*n* = 2). Regarding immunohistochemical staining, all ectopic tumors were positive for ACTH by inclusion criteria. In addition, chromogranin was positive in 20 out of 24 cases (83.3%). Synaptophysin was positive in 22 out of 24 cases (91.7%). Both markers were co-positive in 19 out of 24 cases (79.2%). These findings are consistent with previously reported positivity rates for these markers in neuroendocrine tumors associated with EAS. Patients with EAS were diagnosed at an older age than those with CD (53.63 ± 15.75 years *vs*. 40.45 ± 12.91 years, *P* < 0.001). The proportion of females was significantly lower in the EAS group compared with the CD group (54.2% *vs*. 90.1%, *P* < 0.001). No significant differences were observed in systolic blood pressure, diastolic blood pressure, or body mass index (BMI) between the two groups (Table 1). Metabolic abnormalities were more pronounced in the EAS group. Levels of AST (20.50 (15.25, 28.75) U/L *vs*. 16.00 (13.00, 25.00) U/L, *P* = 0.045) and fasting blood glucose (6.08 (4.70, 8.57) mmol/L *vs*. 5.09 (4.31, 5.77) mmol/L, *P* = 0.012) were significantly higher compared with the CD group. In contrast, LDL, C (2.88 ± 0.87 mmol/L *vs*. 3.39 ± 1.12 mmol/L, *P* = 0.04) and albumin (36.78 ± 4.70 g/L *vs*. 42.54 ± 3.94 g/L, *P* < 0.001) were significantly lower in EAS patients. Electrolyte profiles also differed between groups. Serum potassium (3.04 ± 0.96 mmol/L *vs*. 3.78 ± 0.47 mmol/L, *P* = 0.001), chloride (102.83 ± 5.72 mmol/L *vs*. 106.22 ± 3.10 mmol/L, *P* = 0.001), and calcium (2.14 (2.06, 2.24) mmol/L *vs*. 2.30 (2.27, 2.35) mmol/L, *P* < 0.001) were significantly lower in the EAS group. Conversely, serum phosphorus (0.88 ± 0.22 mmol/L *vs*. 1.05 ± 0.21 mmol/L, *P* = 0.002), 24, hour urinary potassium (103.97 (56.55, 119.58) mmol/24 h *vs*. 52.66 (43.13, 62.03) mmol/24 h, *P* < 0.001), and 24, hour urinary chloride (297.00 (219.75, 375.75) mmol/24 h *vs*. 164.00 (138.00, 195.50) mmol/24 h, *P* = 0.008) were significantly higher in EAS patients compared with those with CD (Table 2). The incidence of hypokalemia (defined as serum potassium <3.5 mmol/L) was significantly higher in the EAS group than in the CD group (66.7% (16/24) *vs*. 11.7% (6/51), $\chi^2$ = 23.731, *P* < 0.001).

**Table 1:** General characteristics of the overall population of patients with Cushing’s syndrome and of each aetiologic group.

	CD group (*n* = 51)	EAS group (*n* = 24)	*P*
Females (%)	90.1% (46/51)	54.2% (13/24)	<0.001*
Age at diagnosis (y)	40.45 ± 12.91	53.63 ± 15.75	<0.001*
SBP (mmHg)	140.24 ± 18.62	143.08 ± 27.56	0.600
DBP (mmHg)	89.47 ± 13.71	86.58 ± 18.33	0.449
BMI (Kg/m^2^)	24.74 (22.92–26.80)	23.90 (22.02–25.98)	0.248

**Note:**

* *P* < 0.05 *vs*. ectopic.

**Table 2:** Comparison of biochemical parameters including liver and renal function, blood glucose, lipids, and electrolytes in patients with Cushing’s disease and ectopic ACTH syndrome.

	CD group (*n* = 51)	EAS group (*n* = 24)	*P*
ALT (U/L)	24.50 (16.00, 37.25)	29.00 (20.00, 63.75)	0.085
AST (U/L)	16.00 (13.00, 25.00)	20.50 (15.25, 28.75)	0.045*
GGT (U/L)	29.00 (20.75, 36.50)	34.00 (23.00, 62.50)	0.188
AKP (U/L)	78.00 (61.75, 103.25)	87.50 (68.00, 101.5)	0.575
Total protein (g/L)	66.15 (61.08, 69.38)	55.90 (50.48, 62.13)	<0.001*
Albumin (g/L)	42.54 ± 3.94	36.78 ± 4.70	<0.001*
Globulin (g/L)	23.17 ± 3.40	19.75 ± 4.33	<0.001*
TC (mmol/L)	5.48 ± 1.33	4.85 ± 1.10	0.054
HDL-C (mmol/L)	1.46 ± 0.34	1.45 ± 0.45	0.930
LDL-C (mmol/L)	3.39 ± 1.12	2.88 ± 0.87	0.040*
TG (mmol/L)	1.28 (0.90, 1.99)	1.17 (0.91, 1.96)	0.763
FBG (mmol/L)	5.09 (4.31, 5.77)	6.08 (4.70, 8.57)	0.012*
Cr (umol/L)	53.00 (43.00, 59.50)	49.00 (41.50, 62.00)	0.690
K (mmol/L)	3.78 ± 0.47	3.04 ± 0.96	0.001*
Na (mmol/L)	144.00 (142.00, 146.00)	146.50 (143.00, 148.00)	0.011*
Cl (mmol/L)	106.22 ± 3.10	102.83 ± 5.72	0.011*
Ca (mmol/L)	2.30 (2.27, 2.35)	2.14 (2.06, 2.24)	<0.001*
P (mmol/L)	1.05 ± 0.21	0.88 ± 0.22	0.002*
Mg (mmol/L)	0.95(0.91, 1.01)	0.93 (0.86, 0.98)	0.050
24-h Urine K (mmol/24 h)	52.66 (43.13, 62.03)	103.97 (56.55, 119.58)	0.009*
24-h Urine Na (mmol/24 h)	214.11 ± 99.21	280.73 ± 132.14	0.094
24-h Urine Cl (mmol/24 h)	164.00 (138.00, 195.50)	297.00 (219.75, 375.75)	0.008*
24-h Urine Ca (mmol/24 h)	6.00 ± 3.12	8.17 ± 3.33	0.085
24-h Urine P (mmol/24 h)	20.42 ± 5.94	15.54 ± 10.73	0.106

**Note:**

* *P* < 0.05 *vs*. ectopic.

### Tumor markers

Levels of several tumor markers were significantly higher in the EAS group than in the CD group, including CYFRA 21-1 (4.22 ± 1.96 U/mL *vs*. 3.03 ± 1.32 U/mL, *P* = 0.017), CEA (4.42 (2.67, 7.83) ng/mL *vs*. 2.53 (1.55, 4.30) ng/mL, *P* = 0.026), CA15-3 (11.50 (7.78, 14.54) U/mL *vs*. 7.47 (5.49, 8.80) U/mL, *P* = 0.004), and CA19-9 (29.70 (16.30, 43.67) U/mL *vs*. 12.90 (9.42, 18.80) U/mL, *P* = 0.002). In contrast, neuroendocrine tumor-related markers, including ProGRP (47.10 (28.50, 87.46) pg/mL *vs*. 32.70 (24.51, 43.51) pg/mL, *P* = 0.065) and NSE (15.60 (14.00, 18.86) ng/mL *vs*. 13.35 (11.30, 16.40) ng/mL, *P* = 0.088), showed no significant differences between the two groups (Table 3).

**Table 3:** Comparison of serum tumor marker levels in patients with Cushing’s disease and ectopic ACTH syndrome.

	CD group (*n* = 51)	EAS group (*n* = 24)	*P*
SCC (ng/mL)	0.62 (0.51, 1.24)	0.60 (0.50, 0.78)	0.218
ProGRP (pg/mL)	32.70 (24.51, 43.51)	47.10 (28.50, 87.46)	0.065
CYFRA 21-1 (ng/mL)	3.03 ± 1.32	4.22 ± 1.96	0.017*
AFP (ng/mL)	3.25 (1.89, 5.14)	3.16 (2.05, 5.14)	0.844
CEA (ng/mL)	2.53 (1.55, 4.30)	4.42 (2.67, 7.83)	0.026*
CA-153 (U/mL)	7.47 (5.49, 8.80)	11.50 (7.78, 14.54)	0.004*
CA-199 (U/mL)	12.90 (9.42, 18.80)	29.70 (16.30, 43.67)	0.002*
CA-125 (U/mL)	18.78 (12.40, 26.20)	22.07 (18.87, 50.31)	0.110
CA-724 (U/mL)	6.70 (1.18, 11.35)	1.78 (0.75, 5.08)	0.066
NSE (ng/mL)	13.35 (11.30, 16.40)	15.60 (14.00, 18.86)	0.088

**Note:**

* *P* < 0.05 *vs*. ectopic.

### Hormone levels and functional tests

Cortisol and ACTH levels at all measured time points were significantly higher in the EAS group than in the CD group. Cortisol levels were elevated at 08:00 (40.79 (25.80, 73.10) μg/dL *vs*. 25.36 (18.77, 33.63) μg/dL, *P* < 0.001), 16:00 (37.47 (23.20, 57.55) μg/dL *vs*. 17.65 (13.60, 21.80) μg/dL, *P* < 0.001), and 24:00 (36.95 (27.62, 56.40) μg/dL *vs*. 17.09 (12.27, 22.43) μg/dL, *P* < 0.001). Similarly, ACTH levels were significantly higher at 08:00 (147.85 (86.65, 302.28) pg/mL *vs*. 84.98 (61.71, 105.30) pg/mL, *P* < 0.001), 16:00 (125.50 (69.06, 256.53) pg/mL *vs*. 57.71 (42.40, 84.40) pg/mL, *P* < 0.001), and 24:00 (142.45 (75.50, 234.40) pg/mL *vs*. 59.70 (36.63, 78.84) pg/mL, *P* < 0.001) (Table 4).

**Table 4:** Comparison of hormone levels in patients with Cushing’s disease and ectopic ACTH syndrome.

	CD group (*n* = 51)	EAS group (*n* = 24)	*P*
Cortisol 8 am (ug/dL)	25.36 (18.77, 33.63)	40.79 (25.80, 73.10)	<0.001*
Cortisol 4 pm (ug/dL)	17.65 (13.60, 21.80)	37.47 (23.20, 57.55)	<0.001*
Cortisol 12 am (ug/dL)	17.09 (12.27, 22.43)	36.95 (27.62, 56.40)	<0.001*
ACTH 8 am (pg/mL)	84.98 (61.71, 105.30)	147.85 (86.65, 302.28)	<0.001*
ACTH 4 pm (pg/mL)	57.71 (42.40, 84.40)	125.50 (69.06, 256.53)	<0.001*
ACTH 12 am (pg/mL)	59.70 (36.63, 78.84)	142.45 (75.50, 234.40)	<0.001*
24 h-UFC (ug/24 h)	476.80 (218.51, 1150.00)	835.50 (361.99, 1558.26)	0.071

**Note:**

* *P* < 0.05 *vs*. ectopic.

### Diagnostic efficacy analysis

ROC curve analysis using the serum cortisol suppression rate after the HDDST showed an AUC of 0.917 (95% CI [0.889–0.945]) for diagnosing EAS. The optimal cut-off value was 0.348 (34.8% suppression), yielding a sensitivity of 83.3%, a specificity of 91.0%, and a Youden index of 0.743. When Midnight Cort × ACTH/1000 was used as the diagnostic indicator, the AUC for identifying EAS was 0.902 (95% CI [0.832–0.972], *P* < 0.001). The optimal cut-off value was 2, with a sensitivity of 83.3%, a specificity of 84.3%, and a Youden index of 0.676 (Table 5). A pairwise comparison of the ROC curves for these two indicators was performed using the DeLong test. The difference between the two AUCs was 0.062 (*Z* = 0.716), which was not statistically significant (*P* = 0.474) (Fig. 1). Bootstrap ROC analysis with 1000 replicates for Midnight Cort × ACTH/1000 yielded a 95% CI [0.821–0.960]. As shown in Fig. 2, the optimism-corrected AUC of Midnight Cort × ACTH/1000 *via* bootstrap resampling remained 0.902, which was fully consistent with the original AUC. Robustness evaluation of the Midnight Cort × ACTH/1000 cut-off value (>2) was performed *via* bootstrap sampling, which showed a mean sensitivity of 0.831 (95% CI [0.652–0.958], SD = 0.079) and a mean specificity of 0.831 (95% CI [0.740–0.936], SD = 0.051).

**Table 5:** Analysis of differential diagnostic efficacy.

	AUC	Optimal cut-off value	Sensitivity (%)	Specificity (%)	95% CI	Youden index
HDDST suppression	0.917	0.348	83.3	91.0	[0.889–0.945]	0.743
Midnight Cort × ACTH/1000	0.902	1.998	83.3	84.3	[0.832–0.972]	0.676

**Figure 1:**
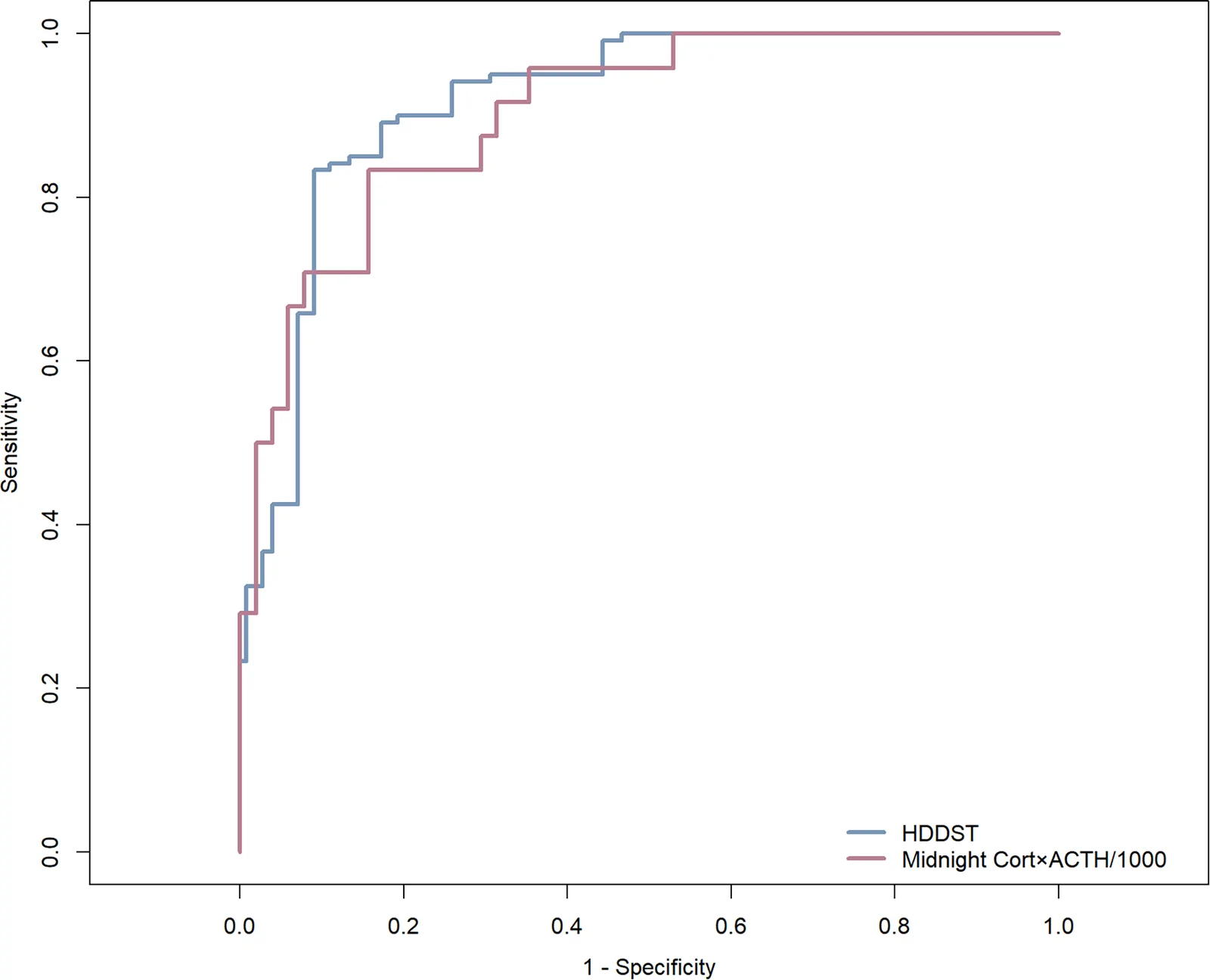
ROC curve of the cortisol suppression rate after HDDST.

**Figure 2:**
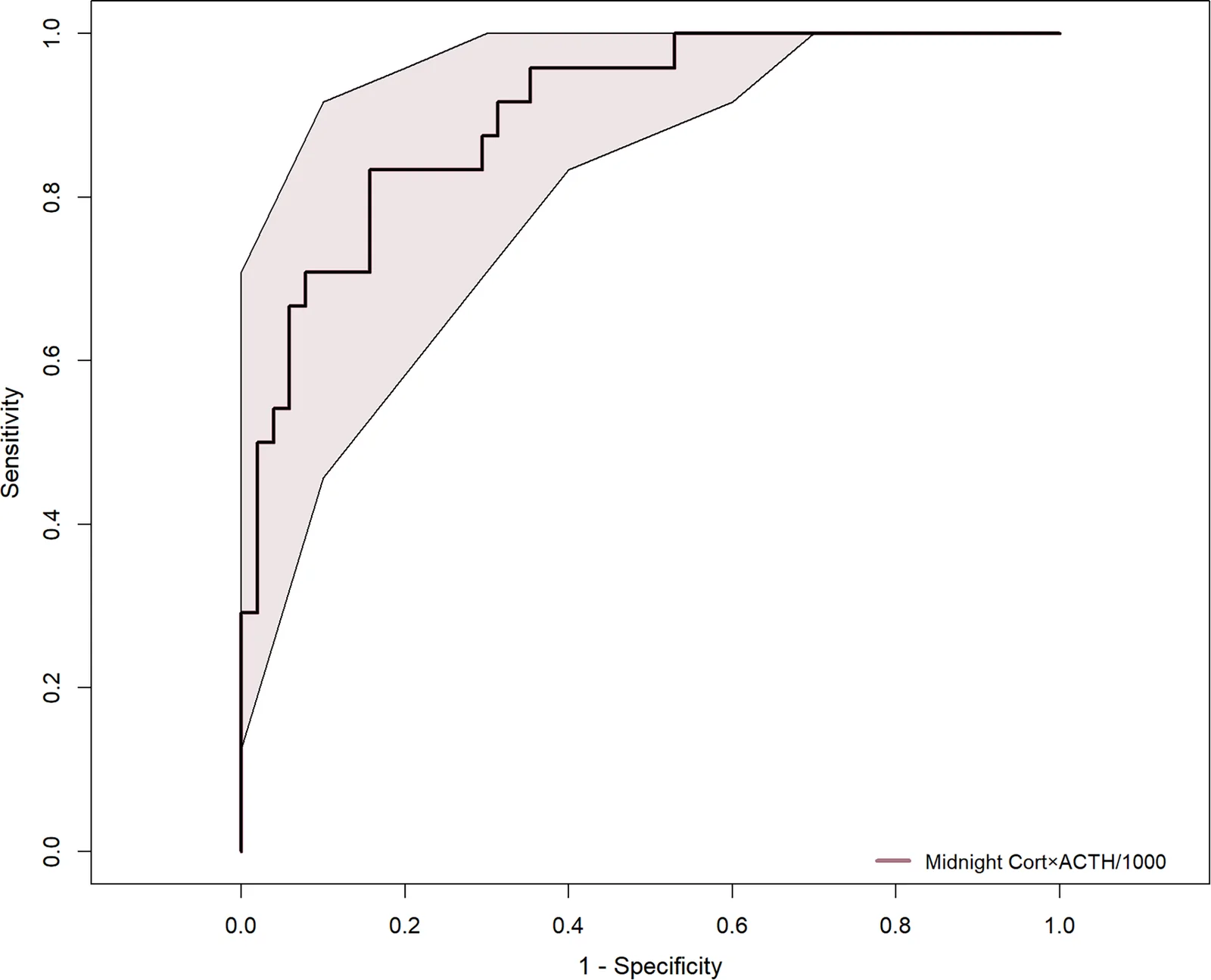
ROC curve of Midnight Cort × ACTH/1000.

## Discussion

Accurate differential diagnosis is essential for determining appropriate treatment strategies and predicting prognosis in CD and EAS. Bilateral inferior petrosal sinus sampling (BIPSS) is regarded as the gold standard for differentiating between the two conditions (Ferriere & Tabarin, 2022), but it is invasive, technically demanding, and not available in all medical centers (Bonelli et al., 2000; Swearingen et al., 2004). Moreover, the procedure carries a risk of false-negative results. Although the high-dose dexamethasone suppression test (HDDST) is commonly used in clinical practice, it requires multiple blood collections, and high-dose corticosteroid exposure may aggravate metabolic disturbances, electrolyte imbalances, and infection risk. Notably, previous studies have shown that the diagnostic performance of HDDST varies considerably in real-world clinical practice. For instance, Attri et al. (2024) recently reported that the test achieved an AUC of only 0.702 when distinguishing EAS from CD. Similarly, Aron, Raff & Findling (1997) found an AUC of just 0.710 for the same purpose. In comparison, the HDDST-based cortisol suppression rate in our study yielded an AUC of 0.917-markedly higher than those earlier estimates. This discrepancy suggests that the diagnostic utility of HDDST may differ substantially depending on the clinical setting, patient population, and testing protocols used. But the lack of domestically produced CRH reagents limits its availability and routine clinical application in China (Fleseriu et al., 2021; Ceccato et al., 2023). Therefore, identifying a simple, non-invasive, and accurate diagnostic approach that can be widely implemented in all levels of medical centers remains an urgent unmet clinical need. Recent studies have indicated that baseline 24-h urinary free cortisol, combined CRH and desmopressin stimulation tests, and imaging assessments offer promising diagnostic value for distinguishing CD from EAS, providing potential new strategies for optimizing diagnostic pathways (Lavoillotte et al., 2024; Frete et al., 2020).

The key finding of this study is the strong diagnostic performance of the novel index “Midnight Cort × ACTH/1000” in distinguishing EAS from CD. This parameter integrates both the absolute concentrations of cortisol and ACTH and their circadian rhythm characteristics. Patients with EAS exhibit markedly elevated cortisol and ACTH levels along with a greater loss of diurnal variation. The product of these two variables accentuates this difference, thereby improving discrimination between the two disorders.

In terms of diagnostic efficacy, the index achieved an AUC of 0.902 in our cohort, which was significantly higher than the previously reported AUC of 0.702 for HDDST in the literature (Attri et al., 2024), and was comparable to the diagnostic performance of standardized HDDST in our center (AUC = 0.917), with no statistically significant difference between the two AUCs (*P* = 0.474). Although the AUC of the index was slightly lower than that of the CRH stimulation test (AUC 0.933–0.975) reported in previous studies (Attri et al., 2024), the index has prominent clinical advantages and incremental value: it only requires a single midnight blood sample, without the need for 48-h high-dose dexamethasone administration, repeated blood sampling, or exposure to exogenous hormone stimulation, thus avoiding the risk of adverse reactions related to hormone administration. Nevertheless, the measurement of midnight serum cortisol is limited by the requirement for venipuncture and in-hospital specimen collection, and it is not recommended as a first-line screening test by the Endocrine Society Clinical Practice Guidelines. In contrast, late-night salivary cortisol (LNSC) enables non-invasive home-based collection, remains stable at room temperature for several weeks, and exhibits favorable sensitivity and specificity for the evaluation of hypercortisolism; notably, LNSC has been endorsed as the first-line screening test for Cushing’s syndrome by the Endocrine Society. Given the superior clinical feasibility of LNSC, future research is warranted to develop and validate a novel diagnostic index based on the product of LNSC and ACTH (LNSC × ACTH) as a more accessible, non-invasive tool for the differential diagnosis of CD and EAS (Attri et al., 2023).

Compared with the traditional HDDST, this index offers greater convenience, requiring only a single blood sample while avoiding potential adverse effects associated with high-dose dexamethasone administration. Although the diagnostic efficacy did not differ significantly from that of HDDST, the new index cannot yet fully replace HDDST or BIPSS. However, it may serve as an effective first-line screening tool for preliminary evaluation. When the value exceeds the threshold (>2), EAS should be strongly suspected, and imaging studies such as chest computed tomography should be performed promptly to identify possible ectopic sources. Future studies should explore combining this index with HDDST results or relevant tumor markers, such as CA19-9, to construct a composite diagnostic model that could further enhance diagnostic accuracy. Validation through larger, multicenter studies will be necessary to confirm its clinical applicability.

In recent years, several noninvasive strategies have been developed to reduce reliance on BIPSS in the differential diagnosis of ACTH-dependent CS. A study from India established a combined noninvasive algorithm using CRH with pituitary MRI and whole-body CT, which achieved 100% positive predictive value for CD and could avoid IPSS in nearly half of candidates (Frete et al., 2020). Meanwhile, a Chinese team constructed a simplified noninvasive scoring model incorporating clinical and biochemical indicators, which exhibited significantly higher diagnostic accuracy (AUC = 0.915) than HDDST (AUC = 0.756) and is particularly suitable for centers without access to BIPSS or CRH reagents (Lyu et al., 2022).

Plasma ACTH and cortisol concentrations at all measured time points were significantly higher in the EAS group than in the CD group, consistent with findings from previous studies (Isidori et al., 2006; Yang et al., 2021). The underlying mechanism reflects differences in feedback regulation within the hypothalamic–pituitary–adrenal (HPA) axis, which also forms the theoretical basis for hormone suppression testing. PitNETs in CD originate from pituitary cells that retain partial sensitivity to glucocorticoid negative feedback (Fleseriu et al., 2021; Nieman et al., 2008), thereby limiting excessive hormone secretion. In contrast, ectopic ACTH-secreting tumors in EAS arise from non-pituitary tissues and completely lose responsiveness to glucocorticoid inhibition (Lacroix et al., 2015; Liddle et al., 1969), resulting in uncontrolled secretion of ACTH and cortisol. This dysregulation contributes to more severe metabolic abnormalities in EAS patients, including hyperglycemia, hypokalemia, and hypoalbuminemia, as observed in the present study.

The marked elevation of non–small cell lung cancer-associated antigen, CEA, CA15-3, and CA19-9 in the EAS group reflects the underlying etiology of this condition. Increased CEA levels are frequently observed in bronchial carcinoids and thymic tumors, elevated CA15-3 is associated with certain thymomas, and raised CA19-9 levels may indicate pancreatic neuroendocrine tumors (Hayes & Grossman, 2018; Ilias et al., 2005; Hammarström, 1999; Li, Xu & Zhang, 2019; Luo et al., 2021). Although the neuroendocrine tumor–related markers ProGRP (*P* = 0.065) and NSE (*P* = 0.088) did not reach statistical significance, both demonstrated a clear upward trend in the EAS group. The marked difference in hypokalemia frequency between EAS (66.7%) and CD (11.7%) in our cohort is consistent with previous reports, where hypokalemia has been identified as a valuable clinical clue favoring EAS, reflecting the more severe mineralocorticoid excess resulting from profound hypercortisolism. Two factors may account for the lack of statistical significance. First, the limited sample size, particularly the relatively small number of EAS cases, likely reduced statistical power. Second, PitNETs in CD, as neuroendocrine tumors themselves, may produce low levels of these markers, thereby diminishing intergroup differences. Despite this, the observed trend indicates that markedly elevated ProGRP or NSE levels in patients with ACTH-dependent CS should raise a strong suspicion of EAS. Future studies with larger cohorts are needed to validate these findings and to establish reliable diagnostic cutoff values for these markers.

Several limitations should be acknowledged. First, the low incidence of CS resulted in a small overall sample size, particularly the limited number of EAS cases. Second, the retrospective study design introduces potential selection bias. Third, the single-center design may limit the generalizability of the findings. The lack of long-term outcome data due to patient attrition represents an important limitation of this study, and future prospective studies with systematic follow-up are needed to clarify the prognostic value of the proposed diagnostic index in EAS patients. Future multicenter, large-sample prospective studies are required to further confirm the diagnostic value of the proposed indicator.

## Conclusion

This study evaluated the diagnostic value of the novel indicator Midnight Cort × ACTH/1000 for differentiating ectopic ACTH syndrome from Cushing’s disease, and compared its efficacy with the high-dose dexamethasone suppression test.

Compared with CD patients, EAS patients had an older diagnostic age, lower female proportion, more severe metabolic and electrolyte disorders, and significantly higher levels of cortisol, ACTH and multiple tumor markers. For differential diagnosis, Midnight Cort × ACTH/1000 achieved an AUC of 0.902, with an optimal cut-off value of 2, a sensitivity of 83.3% and a specificity of 84.3%. Its diagnostic efficacy was comparable to that of HDDST (AUC = 0.917, *P* = 0.474), and bootstrap resampling confirmed the robustness of its diagnostic performance.

This indicator has prominent clinical advantages: it only requires a single midnight blood sample, avoiding the operational inconvenience and hormone-related adverse reactions of HDDST. It can be used as a reliable first-line screening tool for the differential diagnosis of EAS and CD.

## Supplemental Information

10.7717/peerj.21677/supp-1 Supplemental Information 1 Results of Multivariate Logistic Regression Analysis.

10.7717/peerj.21677/supp-2 Supplemental Information 2 Pathological image. The watermark in the image reads ‘病理科打印’ (Pathology Department Print), which is a routine label generated by the hospital pathology system and does not affect data interpretation.

10.7717/peerj.21677/supp-3 Supplemental Information 3 Raw data.

10.7717/peerj.21677/supp-4 Supplemental Information 4 Codebook of the raw data.

## Additional Information and Declarations

### Data Availability

The following information was supplied regarding data availability:

The raw data is available in the Supplemental Files.
