# Supplementary material for: Analysis of biochemical parameters and tumor markers in Cushing’s disease and ectopic adrenocorticotropic hormone syndrome
Source: PeerJ. 2026 Oct 2;14:e21677. doi: 10.7717/peerj.21677 (PMC13637636; doi:10.7717/peerj.21677)

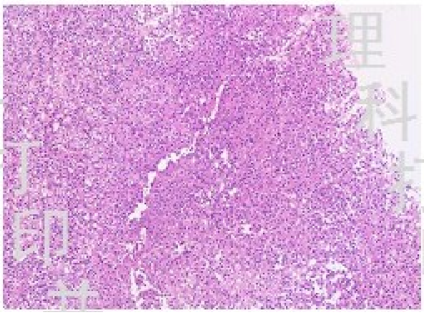

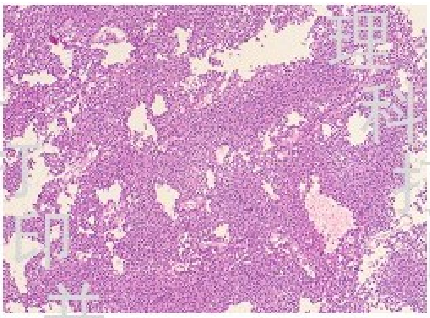

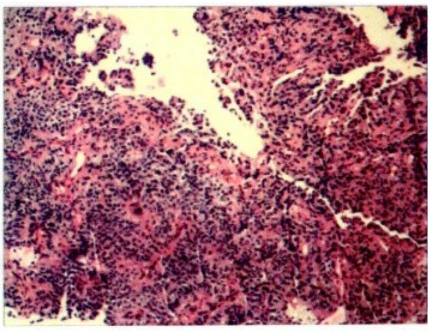

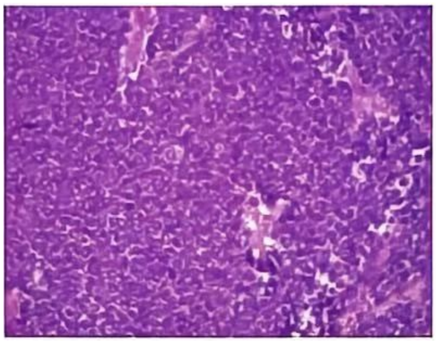

Supplement: Supplementary file 1 [file peerj-14-21677-s002.doc]
