# Supplementary material for: Analysis of biochemical parameters and tumor markers in Cushing’s disease and ectopic adrenocorticotropic hormone syndrome
Source: PeerJ. 2026 Oct 2;14:e21677. doi: 10.7717/peerj.21677 (PMC13637636; doi:10.7717/peerj.21677)
Supplement: Supplementary file 1 [file peerj-14-21677-s001.doc]

|  | B | SE | Wald χ² | P | OR（95% CI） |
| --- | --- | --- | --- | --- | --- |
| Age | -.377 | 293.477 | .000 | .999 | .686(.000-4.414E+249) |
| Glu | 24.946 | 1761.494 | .000 | .989 | 68228768967.582(.000-) |
| Albumin | 3.478 | 1761.494 | .000 | .998 | 32.386(.000-) |
| K | -110.618 | 1144.238 | .000 | .991 | .000(.000-) |
| CYFRA 21-1 | -1.065 | 9611.998 | .000 | 1.000 | .345(.000-) |
| CEA | -29.553 | 3009.313 | .000 | .987 | .000(.000-) |
| CA-153 | 30.808 | 1851.985 | .000 | .985 | 23977763050274.810(.000-) |
| CA-199 | 3.515 | 1684.422 | .000 | .989 | 33.626(.000-) |
| Midnight Cort×ACTH/1000 | -5.585 | 247.277 | .000 | .995 | .004(.000-1.022E+212) |
